# Extracellular DNA Correlates with Intestinal Inflammation in Chemically Induced Colitis in Mice

**DOI:** 10.3390/cells10010081

**Published:** 2021-01-06

**Authors:** Martin Maronek, Barbora Gromova, Robert Liptak, Barbora Konecna, Michal Pastorek, Barbora Cechova, Maria Harsanyova, Jaroslav Budis, David Smolak, Jan Radvanszky, Tomas Szemes, Jana Harsanyiova, Alzbeta Kralova Trancikova, Roman Gardlik

**Affiliations:** 1 Institute of Molecular Biomedicine, Faculty of Medicine, Comenius University in Bratislava, 81108 Bratislava, Slovakia; martin.maronek@gmail.com (M.M.); b.gromova@gmail.com (B.G.); basa.konecna@gmail.com (B.K.); michal.pastorek86@gmail.com (M.P.); 2 Institute of Physiology, Faculty of Medicine, Comenius University in Bratislava, 81372 Bratislava, Slovakia; liptak.robert1@gmail.com; 3 Department of Physiology, Third Faculty of Medicine, Charles University, 10000 Prague, Czech Republic; renaissance256215@gmail.com; 4 Department of Molecular Biology, Faculty of Natural Sciences, Comenius University in Bratislava, 84215 Bratislava, Slovakia; mariavenghova@gmail.com (M.H.); dd.smolak@gmail.com (D.S.); tomasszemes@gmail.com (T.S.); 5 Geneton Ltd., 84104 Bratislava, Slovakia; jaroslav.budis@gmail.com (J.B.); jradvanszky@gmail.com (J.R.); 6 Comenius University Science Park, Univerzita Komenského, 84104 Bratislava, Slovakia; 7 Slovak Centre of Scientific and Technical Information, 81104 Bratislava, Slovakia; 8 Institute of Clinical and Translational Research, Biomedical Research Center, Slovak Academy of Sciences, 84505 Bratislava, Slovakia; 9 Department of Pathophysiology, Jessenius Faculty of Medicine, Comenius University in Bratislava, 03601 Martin, Slovakia; jharsanyiova@gmail.com; 10 Biomedical Center Martin, Jessenius Faculty of Medicine, Comenius University in Bratislava, 03601 Martin, Slovakia; trancikova@gmail.com

**Keywords:** cell-free DNA, ulcerative colitis, neutrophil extracellular traps, deoxyribonuclease activity, PAD4

## Abstract

Circulating extracellular DNA (ecDNA) is known to worsen the outcome of many diseases. ecDNA released from neutrophils during infection or inflammation is present in the form of neutrophil extracellular traps (NETs). It has been shown that higher ecDNA concentration occurs in a number of inflammatory diseases including inflammatory bowel disease (IBD). Enzymes such as peptidyl arginine deiminases (PADs) are crucial for NET formation. We sought to describe the dynamics of ecDNA concentrations and fragmentation, along with NETosis during a mouse model of chemically induced colitis. Plasma ecDNA concentration was highest on day seven of dextran sulfate sodium (DSS) intake and the increase was time-dependent. This increase correlated with the percentage of cells undergoing NETosis and other markers of disease activity. Relative proportion of nuclear ecDNA increased towards more severe colitis; however, absolute amount decreased. In colon explant medium, the highest concentration of ecDNA was on day three of DSS consumption. Early administration of PAD4 inhibitors did not alleviate disease activity, but lowered the ecDNA concentration. These results uncover the biological characteristics of ecDNA in IBD and support the role of ecDNA in intestinal inflammation. The therapeutic intervention aimed at NETs and/or nuclear ecDNA has yet to be fully investigated.

## 1. Introduction

Inflammatory bowel disease (IBD) is characterized as a group of chronic inflammatory diseases such as ulcerative colitis or Crohn’s disease (CD) with long-lasting recurrent inflammation of intestinal epithelium. Since the number of patients increases steadily worldwide, IBD becomes an increasing burden for society [1]. Due to unknown etiopathogenesis, present-day therapy is mainly symptomatic rather than causative. It is also one of the reasons why therapy is often inefficient and costly. Multiple factors have been proposed to contribute to the pathogenesis of IBD such as diet [2], microbial dysbiosis and diversity [3,4] or environment [5].

Extracellular DNA (ecDNA) is by definition any DNA molecule that is present outside of a cell. During cell death, DNA molecules are externalized into the surrounding environment. Therefore, this DNA can be of nuclear or mitochondrial origin. ecDNA is usually present in bodily fluids such as plasma, urine and saliva [6]. Given the fact that cell death usually occurs physiologically (e.g., apoptosis), a certain base-line ecDNA concentration can be measured even under physiological conditions [7,8]. Paradoxically, it appears that ecDNA from any source can be immunogenic because it has been shown to bind the toll-like receptor 9 [9] which in turn causes the activation of the cascade leading to the innate inflammatory response [10,11]. To this reason, ecDNA can be perceived as a marker of inflammation in a number of pathologies and diseases including sepsis [12,13,14], multiple trauma [15] or obesity [16]. In addition, while higher ecDNA concentration was detected in a mouse model of colitis [17,18], single administration of ecDNA from colitic into healthy mice led to amelioration of intestinal inflammation [19,20,21]. This discrepancy further highlights that the role of ecDNA in inflammation has not been adequately described.

In 2004, a new component of innate immunity was discovered. Neutrophils, in addition to phagocytosis, are capable of expelling their DNA along with enzymes and antimicrobial peptides in the outer environment and create net-like traps which serve to immobilize and kill pathogens [22]. These traps are known as neutrophil extracellular traps (NETs) and are formed in a process called NETosis. Despite their unquestionable role in the immune system, excessive NET formation has been observed to exacerbate many pathological states and diseases such as traumatic brain injury [23], liver disease [24], rheumatoid arthritis [25], intestinal barrier function in experimental colitis [26] and they even play a role in tumor microenvironment [27] and colorectal cancer liver metastasis [28].

If higher ecDNA concentration and NET formation causes and/or exacerbates an inflammatory state such as in IBD, then it stands to reason to remove excessive ecDNA and NETs from the circulation. ecDNA is predominantly degraded by the action of endogenous deoxyribonucleases (DNases), enzymes which possess the ability to cleave any DNA molecule. Although the activity of DNases may play a role in IBD, neither they nor ecDNA have gained much attention so far. Our team has already proven that DNase treatment is a promising approach, as it decreased ecDNA concentration in plasma and alleviated septic and inflammatory symptoms in a mouse model of sepsis [29] and thioacetamide-induced hepatorenal injury [30]. Apart from increased NET degradation, it is possible to inhibit their formation. Certain inhibitors which block peptidyl arginine deiminase (PAD), an enzyme crucial to NET formation, act as inhibitors of NETosis because they block citrullination, a vital step in NET formation [31,32].

The aim of this study was to describe the dynamics of NETosis and ecDNA concentration including its fragmentation rate in a mouse model of colitis. Since altered DNase activity has not yet been properly investigated and could account for higher ecDNA concentration, our aim was to elucidate DNase activity both in vitro and in vivo. Additionally, we aimed to discover the effect of NETosis inhibitors on ecDNA concentration and severity of intestinal inflammation.

## 2. Materials and Methods

### 2.1. Animals

In all of the experiments, 8–10 week-old C57BL/6 mice (Charles River, Wilmington, MA, USA) were used. The animals were housed 6 per cage, at a temperature of 23 °C, 50% humidity, with 12/12-h light/dark cycle and were provided with ad libitum access to standard chow and water. Two weeks prior to the experiments, animals were habituated to these conditions. All of the experiments were approved by the State Veterinary and Food Administration of the Slovak Republic (decision 3041/17-221/3) and by the Ethics committee of the Comenius University in Bratislava, Slovak Republic (decision 25 August 2017).

### 2.2. Induction of Colitis

Mice were randomly divided into four groups (one control group (CTRL) and three experimental groups (DSS 3, DSS 5, DSS 7)). Murine model of colitis was induced with 3% dextran sulfate sodium dissolved in tap water (molecular weight = 40,000, AppliChem, Darmstadt, Germany). The number of each experimental group indicates the number of days animals ingested DSS solution, i.e., 3, 5 or 7 days (for DSS 3, DSS 5 and DSS 7, respectively). Mice in the CTRL group received tap water during the entire experiment. The experiment lasted for 7 days and on the last day animals were euthanized.

#### Administration of PAD4 Inhibitors

Cl-amidine (Sigma-Aldrich, St. Louis, MO, USA) and streptonigrin (Sigma-Aldrich, St. Louis, MO, USA) were administered via intraperitoneal injection once daily on days 1 to 5 in the dose of 10 mg/kg and 0.4 mg/kg, respectively, in 100 μL of phosphate buffered saline (PBS).

### 2.3. Assessment of Colitis Severity

Bodyweight and water intake were monitored daily. Stool consistency was scored on a scale from 0 to 3, representing as follows: 0 = thick, formed stool, blood not present; 1 = soft stool, blood not present; 2 = watery stool, blood not present; and 3 = soft or watery stool with the presence of blood. Mice were also macroscopically assessed for the presence of altered behavior.

### 2.4. Isolation of Samples

On day 7 of the experiment, mice were anesthetized with a solution consisting of ketamine (100 mg/mL), xylazine (20 mg/mL) and physiological solution in a 2:1:1 ratio. Blood was collected via retroorbital puncture using ethylenediaminetetraacetic acid (EDTA)- and heparin-coated tubes (Sarstedt, Nűmbrecht, Germany). Plasma for MPO and TNF-α measurement was obtained by centrifugation of blood samples 1600× *g* for 10 min and for ecDNA isolation for 1600× *g* for 10 min followed by 16,000× *g* for 10 min. During collection, tissues were washed thoroughly with fresh cold 0.9% PBS. Tissue samples were snap-frozen in liquid nitrogen immediately after collection and stored at −20 °C until further use. Before the collection of colon samples, colon was excised from ileocecal junction to proximal rectum and colon length was measured.

### 2.5. Protein Concentration Measurement

Concentration of proteins in the tissue homogenate was measured using bicinchoninic acid kit (Sigma-Aldrich, St. Louis, MO, USA) according to manufacturer instructions. Protein concentration was calculated based on the standard calibration curve and was reported as mg of protein.

### 2.6. Colonic Explant Preparation

A 0.5 cm long samples of distal colon were cut longitudinally, washed twice in PBS and put into 1 mL of cultivation medium consisting of RPMI 1640 (Biosera, Nuaille, France), protease inhibitor cocktail (Sigma-Aldrich, St. Louis, MO, USA) and antibiotic-antimycotic solution (Biosera, Nuaille, France). Colon explants were incubated in 24-well plates at 37 °C for 20 h. Afterwards, media were pipetted into Eppendorf tubes and kept frozen until the measurement of ecDNA concentration.

### 2.7. Animal Endoscopy

Prior to endoscopic visualization, animals were fasted overnight. The next day (day 7), animals were anesthetized via intraperitoneal injection of ketamine and xylazine solution, as described in Section 2.4. Before the insertion of rigid endoscope Tele Pack Vet X LED RP 100 (Karl Storz, Tuttlingen, Germany), any remaining stool was removed with massaging the anus. The endoscope was inserted 8 cm proximally into the anus. Epithelium of the colon was examined in 4 categories: Vascular translucency, presence of fibrin deposits, bleeding and reddening of the colon mucosa. Score evaluation was done according to Table 1. The examination was done in a blinded manner. After every examined animal the endoscope was washed first with tap water to remove any residual stool fragments or blood and subsequently with ethanol. Final score was calculated as the sum of each of the examined categories for any given animal.

### 2.8. ecDNA Isolation and Measurement

ecDNA was isolated from the plasma samples using the QiaAmp^®^ DNA Blood Mini Kit (Qiagen, Hilden, Germany) according to the protocol of the manufacturer. The concentration of isolated ecDNA was measured using a Qubit 3.0 fluorometer and Qubit dsDNA HS assay kit (Thermo Fisher Scientific, Waltham, MA, USA). ecDNA was kept frozen at −20 °C until further use.

### 2.9. Real-Time PCR

ncDNA and mtDNA were estimated using real-time PCR on the Mastercycler realplex 4 (Eppendorf, Hamburg, Germany) with SsoAdvanced Universal SYBR Green Supermix master mix (Bio-Rad, Hercules, CA, USA). The PCR reaction was performed according to Malik et al. [33,34]. Primer sequences for ncDNA were: Forward 5′-ATG GGA AGC CGA ACA TAC TG-3′ and reverse 5′-CAG TCT CAG TGG GGG TGA AT-3′ with 177 bp product size. Primer sequences for mtDNA were: Forward 5′-CTA GAA ACC CCG AAA CCA AA-3′ and reverse 5′-CCA GCT ATC ACC AAG CTC GT-3′ with 125 bp product size. The following PCR program was used: One cycle of 95 °C for 5 min (denaturation step), 35 cycles of 95 °C for 15 s for denaturation, 60 °C for 30 s for annealing and polymerization, 95 °C for 5 s for melting, 65 °C for 60 s and continued with 95 °C for one cycle of melt curve analysis. The ecDNA of both a nuclear and mitochondrial origin was expressed in genome equivalents (GE) per ml of plasma.

### 2.10. MPO Activity Measurement

The MPO activity assay in colon homogenates was performed using an in-house assay according to Kim et al., 2012 [35,36].

### 2.11. TNF-α Measurement

The concentration of TNF-α in plasma was evaluated by Mouse TNF-α Quantikine ELISA kit according to manufacturer instructions (R&D Systems, Minneapolis, MN, USA).

### 2.12. Determination of DNase Activity

DNase activity in plasma and explant medium was measured by the modified single radial enzyme diffusion method [21] with the GoodView Nucleic Acid Stain (SBS Genetech, Beijing, China). One microliter of the samples were pipetted into the holes of 1% agarose gel (20 mM Tris-HCl pH 7.5, 2 mM CaCl_2_, 2 mM MgCl_2_ and DNA (5 mg/mL) isolated from chicken livers) and incubated overnight (16–20 h) at 37 °C in the dark and visualized by iBOX (Vision works LP Analysis Software, UVP, Upland, CA, USA). The radius of the circles formed was measured using ImageJ software (NIH, Bethesda, Maryland, MD, USA) and compared to the calibration curve from the DNase standards which were twofold dilutions of DNase I in RDD buffer presented in the set (RNase-free DNase set, Qiagen, Hilden, Germany) with known DNase activity. DNase activity was recalculated and expressed in Kunitz units (KU) per ml or mg of proteins for plasma or explants, respectively.

### 2.13. Flow Cytometry

For analysis of ecDNA that originates from neutrophils, 100 µL of peripheral whole blood was subjected to erythrocyte lysis with ammonium chloride, washed and the remaining pellet was resuspended in previously titrated staining mix consisting of 100 nM Sytox Green (Thermo Fisher Scientific, Waltham, MA, USA) and 0.5 µg/mL of a APC-Ly-6G (Biolegend, San Diego, CA, USA) in RPMI1640 (Biosera, Nuaille, France). Samples were incubated for 15 min at room temperature in the dark and immediately assayed on a BD FACSCanto II (BD Biosciences, Franklin Lakes, NJ, US) flow cytometer and analyzed by FCSExpress 6.0 software (De Novo Software, CA USA). Gating strategy used was to first select neutrophils by Ly6G expression, without accounting for forward scatter/side scatter properties that are heterogenous for NETs in suspension. Peripheral blood granulocytes were then gated according to forward and side scatter so no debris was included in the analysis SytoxGreen positive cells were identified as dead, possibly subjected to NETosis. A minimum of 10,000 cells per condition were analyzed. Representative plot is shown on Figure S1.

### 2.14. Massively Parallel Sequencing of Plasma ecDNA

The DNA library was prepared according to the TruSeq Nano standard protocol (Illumina, San Diego, CA, USA) starting from the end repair step (30 μL starting volume). Briefly, 50 μL of the solution after the end repair step was purified with Sample Purification Beads by adding 100 μL of undiluted magnetic beads. Sample multiplexing was used according to the TruSeq Nano high throughput scheme. After A-tailing and index ligation, the library was purified with Sample Purification Beads twice by adding 1.0× volume in two separate purification steps. Subsequently, the library was amplified using 8 cycles. The amplified library was purified with Sample Purification Beads by adding 1.0× volume once. The final libraries were quantified using the Qubit 3.0 Fluorometer (Life Technologies, Carlsbad, CA, USA) and Qubit dsDNA HS assay kit (Invitrogen, Eugene, OR, USA) and quality checked on the 2100 Bioanalyzer (Agilent Technologies, Waldbronn, Germany) with use of the High Sensitivity DNA analysis kit (Agilent Waldbronn, Germany). The libraries were normalized to 4 nmol·L^−1^, and all samples were pooled together and denatured according to the standard protocol. The final library pool was analyzed on NextSeq system using NextSeq 500/550 High Output Kit v2.5 (150 cycles) (Illumina, San Diego, CA, USA) with pair-end run setting of 2 × 75 bp cycles.

### 2.15. Bioinformatics Processing

Adapters and low-quality ends of sequenced reads were removed using Trimmomatic [37,38] based on quality control statistics generated by FastQC [39,40]. After trimming, fragments without sufficient length of both reads (<35 bp) were removed from the data set. Deduplication was carried out by FastUniq [41,42]. Preprocessed sequenced data were mapped against both the mouse (GRCm38.p6) and the human reference genome (*GRCh38*) using Bowtie2 [43,44]. In the remaining dataset bacterial and viral reads were identified by Kraken2 [45,46] with MiniKraken2_v2 database.

### 2.16. Whole-Mount Immunostaining of the Colon

The whole colons were harvested (from caecum to rectum) and fixed stretched in order to thin the gut wall to allow confocal image throughout the whole thickness of the gut wall. The gut was then cut open along the long axis, pinned on Sylgard-lined dish mucosa upward facing, and fixed in 4% formaldehyde at 4 °C overnight. The tissue was then unpinned and rinsed free floating 3–4 times a day for 2–3 days at 4 °C in PBS. The immunostaining of the gut tissues was performed in 2 mL tubes in order to expose both mucosal and adventitial surfaces to antibodies. In order to minimize the possibility that a portion of the tissue will adhere to the tube wall, the tissues were frequently manipulated (repositioned) with blunt forceps to expose all surfaces. The washing in 1× PBS between staining steps was performed in 50 mL tubes on the rotor at 4 °C. The tissues were permeabilized in 1% Tween 20 (Sigma-Aldrich, St. Louis, MO, USA) diluted in 1× PBS at room temperature for 6 h, washed in 1× PBS (3 times 20 min) using rotator, incubated with Avidin solution from Avidin/Biotin kit (SP-2001, Vector Laboratories, Burlingame, CA, USA) diluted 1:12.5 (4 drops = 80 µL to 1000 µL) in 1% bovine serum albumin in PBS (1% PBS/BSA), washed in 1× PBS (5 times 20 min on rotator). Permeabilized and blocked tissues were incubated in rabbit anti-PAD4 primary antibody (cat. number GTX113945, GeneTex, Irvine, CA, USA) diluted 1:200 in 1% PBS/BSA and Biotin solution from Avidin/Biotin kit (SP-2001, Vector Laboratories, Burlingame, CA, USA) for 48 h at 4 °C (repositioned 5–6 times during incubation), washed in 1× PBS (10 times 30 min on rotator), incubated with goat biotin-XX conjugate anti-rabbit IgG (H + L) secondary antibody (cat. number B2770, Thermo Fisher Scientific, Waltham, MA, USA) diluted 1:100 in 1% PBS/BSA overnight at RT, washed in 1× PBS (10 times 20 min on rotator), incubated with streptavidin, Alexa Fluor^®^ 647 conjugate (cat. number S21374, Thermo Fisher Scientific, Waltham, MA, USA) diluted 1:100 in 1× PBS for 5 h at RT in dark, washed in 1× PBS (3 times 20 min on rotator), incubated in anti-fade (pH 8.6) glycerol (Sigma-Aldrich, St. Louis, MO, USA) for 24 h at RT, and stored in anti-fade glycerol at 4 °C. The gut specimen was placed either muscle or mucosal side up on a glass slide and covered with coverslip (24 by 50 mm).

Zeiss Axio Examiner microscope with LSM 880 confocal system and argon laser 633 nm was used for imaging (Carl Zeiss, Jena, Germany). In order to investigate the structures throughout the whole thickness and whole area of the tissue, the complete image of the whole tissue was acquired. This was accomplished by using Tile and Z-stack modules of Zen software. The whole area of the sample was divided into individual square tiles by using the Tile function (each tile was the size of a single visual field that was determined by the properties of the objective used) and for each tile confocal optical sections spaced 1 μm were obtained throughout the whole thickness of the tissue. In all experiments, 20× objective (Zeiss Plan-Apochromat 20×/0.8 M27, Carl Zeiss, Oberkochen, Germany) with a single visual field of 424.9 μm by 424.9 μm was used and the image was acquired with the resolution of 2048 pixels by 2048 pixels. Zeiss Zen software was used for image acquisition and processing.

### 2.17. Statistical Analysis

Data were analyzed using either one- or two-way ANOVA and a post-hoc test. Data are presented as mean ± standard deviation. *p*-values less than 0.05 were considered statistically significant. All analyses were performed using GraphPad Prism 6 Software (GraphPad Software, La Jolla, CA, USA). All statistical analyses of sequenced data were performed with in-house scripts in Python. Distribution of fragments lengths mapped to the mouse genome we computed as a relative abundance of fragments of given lengths over individual samples. PCA was calculated using the Scikit-learn library. The input dataset was a standardized distribution of fragment lengths in the samples. Proportion of reads from different types of organisms was computed as the normalized number of reads for the deduplicated dataset. Data visualization was performed by Matplotlib and Seaborn library.

## 3. Results

### 3.1. Dextran Sulfate Sodium (DSS) Induced Intestinal Inflammation

Ingestion of DSS solution led to weight loss starting from day three in the DSS 7 group. Animals in the DSS 5 group began to lose weight on day five and average body weight of the DSS 3 group on day seven was lower compared to the control (CTRL) group (Figure S2A). In general, longer DSS ingestion caused worse signs of intestinal inflammation. Since water intake is a voluntary activity, DSS consumption for each animal may vary and may account for observed differences. To this reason, daily water intake was measured. No significant differences among groups were detected (data not shown).

A rise in the stool consistency score (manifested as presence of softer stool) was observed in the DSS 7 group on day one and on day four blood was detected (Figure S2B). DSS 5 group showed blood in the stool on day six and DSS 3 group did not exhibit any intestinal bleeding throughout the entire experiment. Compared to the CTRL group, each experimental group showed an average stool consistency score above one. Colon length confirmed extensive inflammation as well. The average colon length of the CTRL group was significantly longer compared to both DSS 5 (*p* < 0.001) and DSS 7 (*p* < 0.001) groups (Figure S2C). There was no difference in colon length between CTRL and DSS 3 groups. Intestinal inflammation was confirmed with increased myeloperoxidase (MPO) activity in DSS7 compared to CTRL (*p* < 0.001, Figure S2D). The concentration of tumor necrosis factor α (TNF-α) showed a rising trend with the highest value on day five and decreased on day seven (*p* = 0.003, Figure S2E).

On day seven, prior to euthanasia, two animals from each group were endoscopically examined. Inflammation of the colon wall manifests as reddening, swelling, fibrin deposition, invisible vasculature and bleeding. While animals from the CTRL group did not show any signs of inflammation (Figure S3A), worse outcome was seen in all experimental groups (Figure S3B–D). During examination, animals were assigned a total endoscopy score (Figure S3E). Taken together, endoscopic assessment confirmed that the mouse model of colitis was induced, and its severity exhibited a time-dependent effect.

### 3.2. Total ecDNA Is Increased in Plasma but Not in Colon Explant Medium

**Figure 1:**
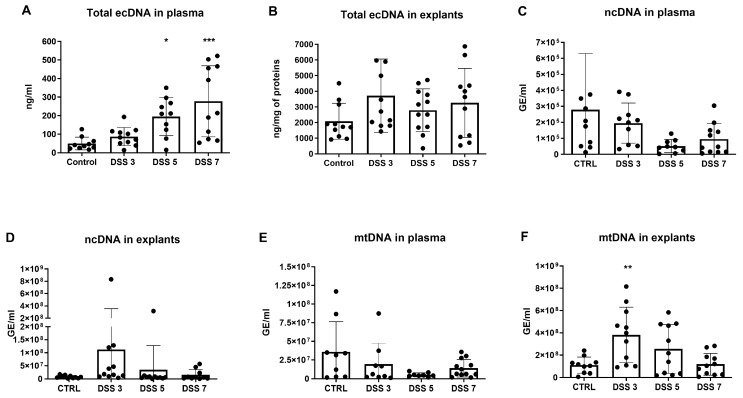
Total, nuclear and mitochondrial DNA in plasma and colon explants. (**A**) the concentration of total ecDNA in plasma showed a time-dependent trend and was highest in DSS 7 group. (**B**) total extracellular DNA (ecDNA) in explants rose on day 3, however was lower in other timepoints. (**C**) the concentration of nuclear (ncDNA) in plasma did not differ between the groups. (**D**) ncDNA in explants reached the highest amount on day 3 and subsequently decreased until the end of the experiment. (**E**) mitochondrial (mtDNA) in plasma did not differ between the groups. (**F**) mtDNA in explants showed a similar pattern compared to ncDNA in explants, where mtDNA concentration rose on day 3 and decreased in other timepoints. * = *p* < 0.05; ** = 0.05 > *p* > 0.01; *** = *p* < 0.001.

By comparing CTRL with the experimental groups, concentration of the total ecDNA in plasma showed a rising trend with increased disease severity, from 1.7-fold increase in the DSS 3 group to 5.5-fold increase in the DSS 7 group (*p* < 0.001, Figure 1A). In total ecDNA in explant medium, DSS 3 group showed the highest concentration (1.8-fold higher compared to CTRL group) while the concentrations of other experimental groups were similar to CTRL group (Figure 1B).

Since total ecDNA can be divided based on its origin into nuclear (ncDNA), mitochondrial (mtDNA) and microbial, ncDNA and mtDNA in both plasma and explant medium was measured. The concentrations of both ncDNA and mtDNA in plasma of all DSS groups did not show any significant difference compared with CTRL group (Figure 1C,E). Although not significant, the amount of ncDNA in explant medium was the highest in the DSS 3 group and there was a decreasing trend in other experimental groups (Figure 1D). In case of mtDNA in explant medium, the same pattern was observed with the highest concentrations in DSS3 group (*p* = 0.003, Figure 1F).

**Figure 2:**
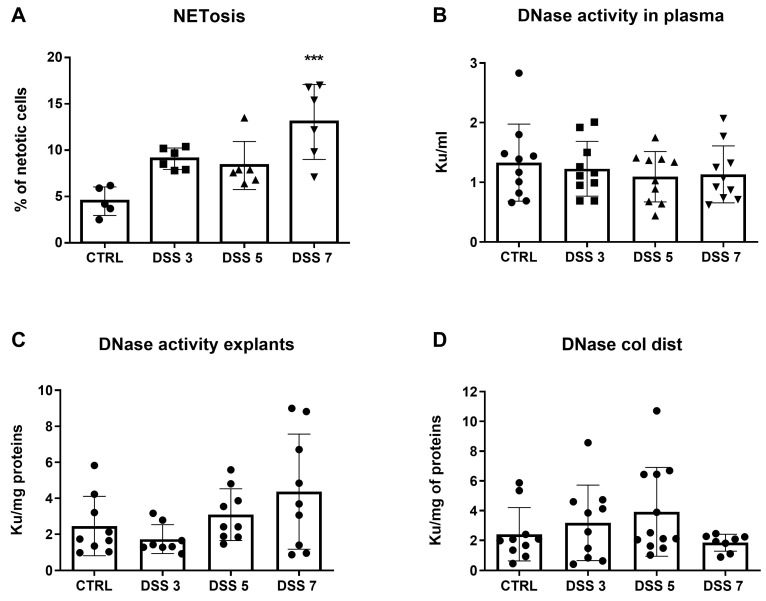
Neutrophil extracellular trap (NET) osis and deoxyribonuclease (DNase) activity. (**A**) percentage of cells undergoing NETosis was elevated in both DSS 3 and DSS 5 groups compared to control group, however the highest percentage was observed in DSS 7 group. (**B**) DNase activity in plasma did not differ between the groups. (**C**) DNase activity in explants showed a rising trend along with increasing disease severity. (**D**) DNase activity in the distal colon was the highest on day 5, however decreased on day 7. *** = *p* < 0.001.

As NETosis seems to be a major source of circulating ecDNA, we decided to analyze the amount of NET-otic cells. Flow cytometry revealed a 2.9-fold higher percentage of cells undergoing NETosis in DSS 7 group compared to CTRL group (*p* < 0.001, Figure 2A). The concentration of total ecDNA could be at least partially explained by the activity of DNases, the enzymes which specifically cleave DNA. DNase activity in plasma, explant medium and the distal part of the colon was measured. Interestingly, DNase activity in plasma did not show any differences among groups (Figure 2B). However, DNase activity in explants was the lowest in DSS3 and the highest in DSS7 group (*p* = 0.06 Figure 2C). This could partially explain the pattern of ncDNA and mtDNA in colon explants. DNase activity in the distal colon showed a slightly increasing trend up to day five but was the lowest on day seven (Figure 2D).

**Figure 3:**
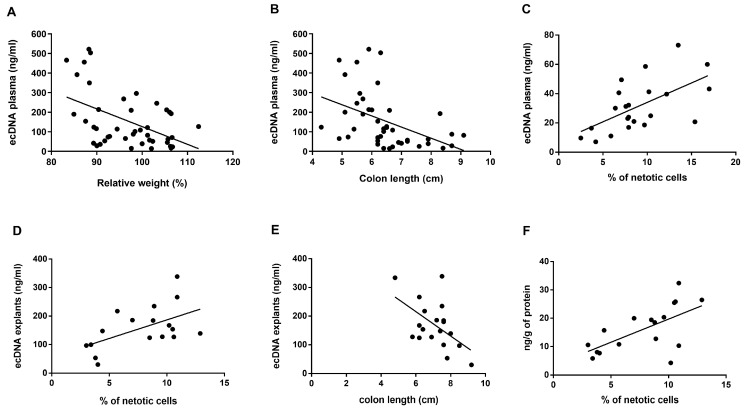
Correlations of total ecDNA and disease markers. The concentration of total ecDNA in plasma correlated with (**A**) relative weight of mice (*p* = 0.001, r^2^ = 0.2), (**B**) colon length (*p* = 0.0025, r^2^ = 0.2) and (**C**) percentage of cells undergoing NETosis (*p* = 0.003, r^2^ = 0.36). The concentration of total ecDNA in explants correlated with (**D**) percentage of cells undergoing NETosis (*p* = 0.025, r^2^ = 0.29) and (**E**) colon length (*p* = 0.019, r^2^ = 0.28). (**F**) the concentration of TNF-α correlated with percentage of cells undergoing NETosis.

Further, we correlated the observed ecDNA levels in plasma and explants with disease activity markers. Plasma ecDNA negatively correlated with weight (*p* = 0.001, r^2^ = 0.23, Figure 3A) and colon length of the mice (*p* = 0.0025, r^2^ = 0.2, Figure 3B). Positive correlations were found between the percentage of cells undergoing NETosis and the concentration of total ecDNA in plasma (*p* = 0.003, r^2^ = 0.36, Figure 3C) and total ecDNA in explants (*p* = 0.025, r^2^ = 0.29, Figure 3D). In addition, concentration of total ecDNA in explants negatively correlated with colon length (*p* = 0.019, r^2^ = 0.28, Figure 3E). Last, the levels of TNF-α positively correlated with percent of cells undergoing NETosis (*p* = 0.007, r^2^ = 0.39, Figure 3F).

### 3.3. Specific Fragmentation and Origin of Plasma ecDNA Related to Colitis Severity

**Figure 4:**
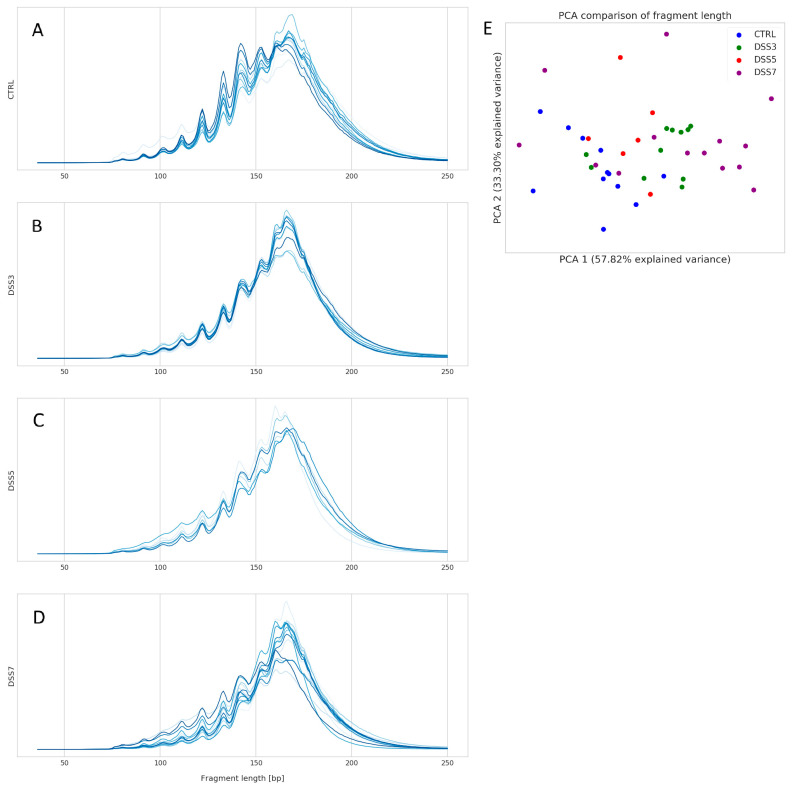
Distribution of ecDNA fragments. Size and relative abundance of plasma ecDNA fragments in (**A**) CTRL, (**B**) DSS3, (**C**) DSS5 and (**D**) DSS7. CTRL shows higher abundance of fragment size 120–160 bp compared with other groups. Intragroup variability in the number of specific fragment sizes rises from CTRL towards DSS7 group. DSS7 showed the highest variability of fragment sizes and relative abundance. (**E**) principal component analysis (PCA) comparison of fragment sizes shows that the groups do not form bordered clusters. However, a clear left-to-right trend on *x*-axis is obvious from CTRL to DSS7 group.

Fragment analysis was performed on total plasma ecDNA. The distribution of ecDNA fragments sizes did not show an obvious group-specific pattern. However, CTRL group has highest abundance of fragment sized 120–160 bp among all groups (Figure 4A). The variability of fragment distribution is higher in the groups with colitis compared with CTRL, with an increasing trend towards DSS7 group (Figure 4B–D). The most inconsistent distribution of fragments can be seen in DSS7.

The relatively high variability of ecDNA fragments is shown on principal component analysis (PCA) plot (Figure 4E). The groups do not form separate clusters; however, a clear left-to-right trend of group clustering can be seen on *x*-axis. Interestingly, DSS3 samples are closer to DSS7 than to DSS5.

**Figure 5:**
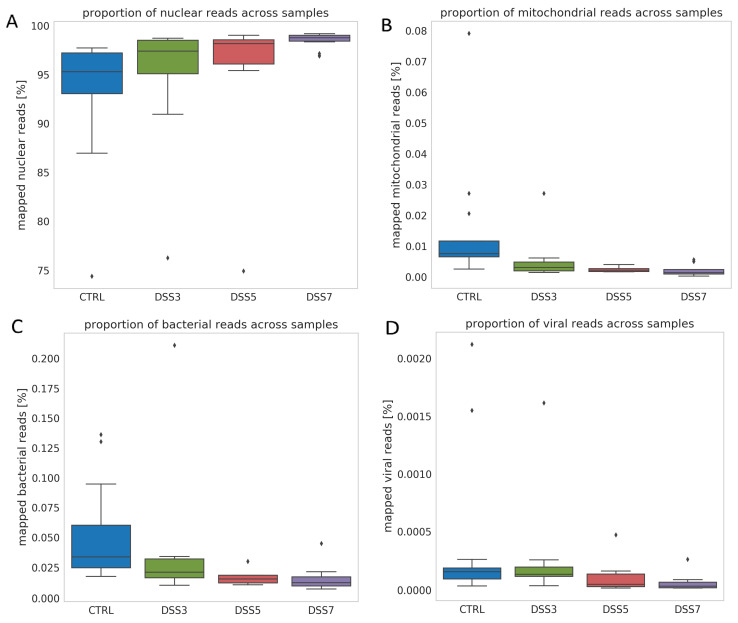
Origin of ecDNA. (**A**) Relative proportion of mapped nuclear reads shows an increasing trend from CTRL toward DSS7. Relative proportion of (**B**) mitochondrial, (**C**) bacterial and (**D**) viral mapped reads shows decreasing trend from CTRL towards DSS7 group. Graph shows proportion of reads relative to total number of mapped reads.

Further, relative proportions of mapped reads belonging to nuclear, mitochondrial, bacterial and viral genomes were analyzed. Relative proportion of (A) nuclear DNA shows an increasing trend from CTRL toward DSS7. Relative proportion of (B) mitochondrial, (C) bacterial and (D) viral mapped reads shows decreasing trend from CTRL towards DSS7 group. Graph shows proportion of reads relative to total number of mapped reads. Relative proportion of mapped nuclear reads shows an increasing trend from CTRL toward DSS7 (Figure 5A). Contrary to this, relative proportion of mitochondrial (Figure 5B), bacterial (Figure 5C) and viral (Figure 5D) mapped reads shows decreasing trend from CTRL towards DSS7 group.

### 3.4. PAD4 Is Increased in Colonic Tissue of Mice with Severe Colitis

**Figure 6:**
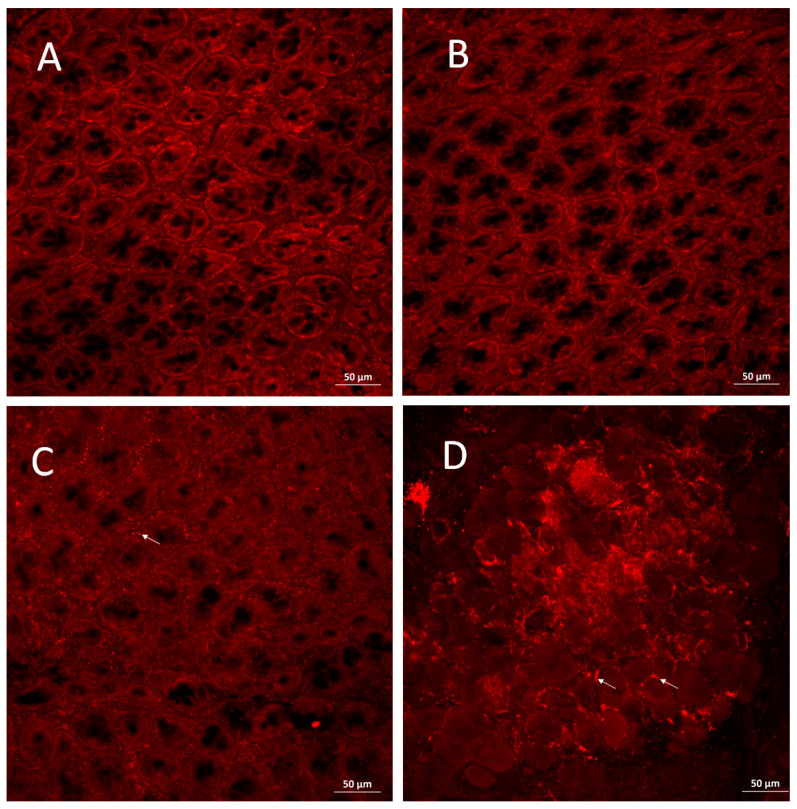
Immunostaining of colonic mucosa. Crypts in (**A**) CTRL and (**B**) DSS3 group show no sign of damage and no expression of PAD4. (**C**) colonic mucosa in DSS5 group is damaged and with peptidyl arginine deiminase (PAD)4-specific signal at the crypt borders. (**D**) colonic mucosa crypts in DSS7 group are disorganized and with strong PAD4-specific signal. Arrows show locations of PAD4-specific signal.

The presence of PAD4 protein in the colonic tissue was analyzed using whole-mount immunostaining. Images show that the mucosal layer is well organized in CTRL and DSS3 groups with no specific signal representing the PAD4 protein (Figure 6A,B). Colonic mucosa in DSS5 group is slightly disorganized, as seen on the altered shape of the crypts. In addition, a PAD4-specific signal is shown along the borders of the damaged crypts (Figure 6C). Colonic mucosa in DSS7 is largely disorganized with no properly shaped crypts and strong PAD4-specific signal in the damaged mucosal layer (Figure 6D).

### 3.5. Inhibition of NETosis Partially Ameliorated Colitis

Cl-amidine [33,47] and streptonigrin [35,48] have been previously reported as PAD4 inhibitors which helped alleviate intestinal inflammation in a mouse model of colitis [34,36,37,39]. Animals were administered with these inhibitors and the concentration of total ecDNA, ncDNA and mtDNA was analyzed.

**Figure 7:**
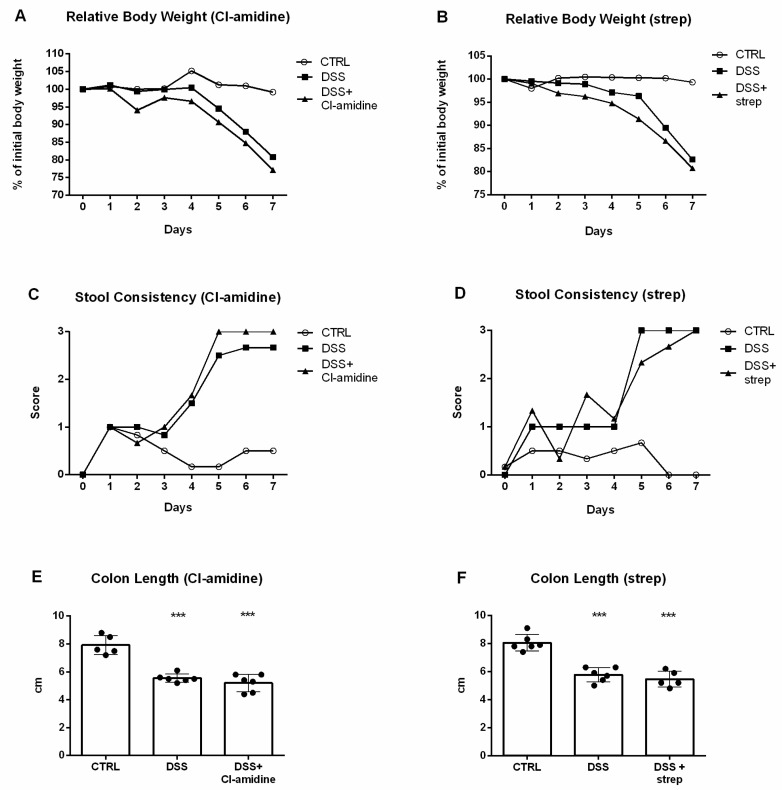
Comparison of Cl-amidine and streptonigrin treatment. Administration of either Cl-amidine or streptonigrin did not lead to amelioration of weight loss (**A**,**B**), stool consistency score (**C**,**D**) or colon length (**E**,**F**) *** = *p* < 0.001.

Application of either Cl-amidine or streptonigrin did not lead to any improvement in body weight (Figure 7A,B), stool consistency (Figure 7C,D) or colon length (Figure 7E,F).

**Figure 8:**
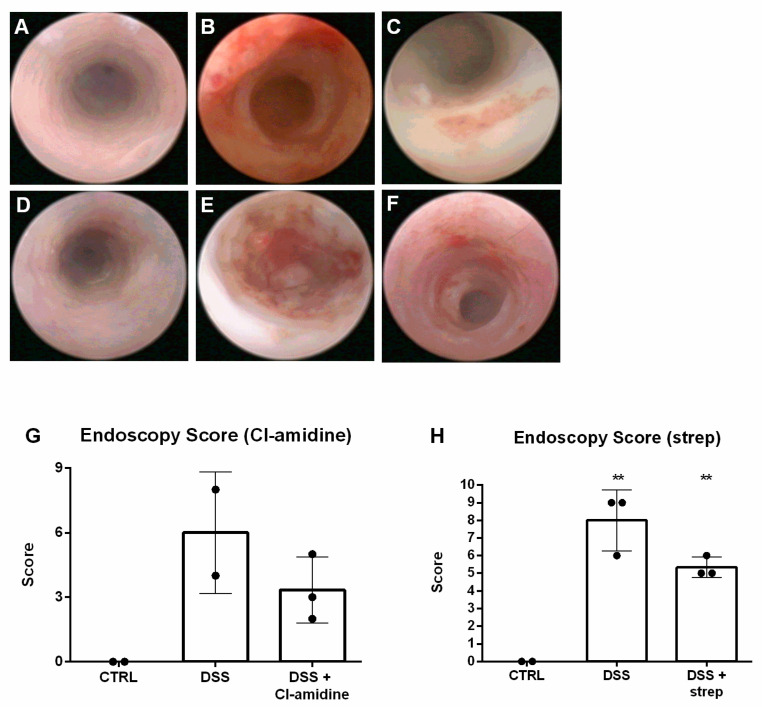
Representative images of endoscopic visualization of the colonic mucosa. When compared to negative (**A**) and positive control (**B**), administration of Cl-amidine visibly alleviated intestinal inflammation (**C**). Application of strep (**F**) managed to partially suppress the inflammation as well compared to negative (**D**) and positive control (**E**). Endoscopy score confirmed the partial amelioration of both Cl-amidine (**G**) and strep (**H**) treatment. ** = 0.05 > *p* > 0.01.

Despite this, endoscopy revealed partial amelioration in both Cl-amidine (Figure 8C) and streptonigrin (Figure 8F) treated DSS groups compared to healthy mice (CTRL) and untreated mice with colitis (DSS) (Figure 8A,B for Cl-amidine and Figure 8D,E for streptonigrin, respectively). The observed partial amelioration was confirmed by endoscopic score (Figure 8G,H).

**Figure 9:**
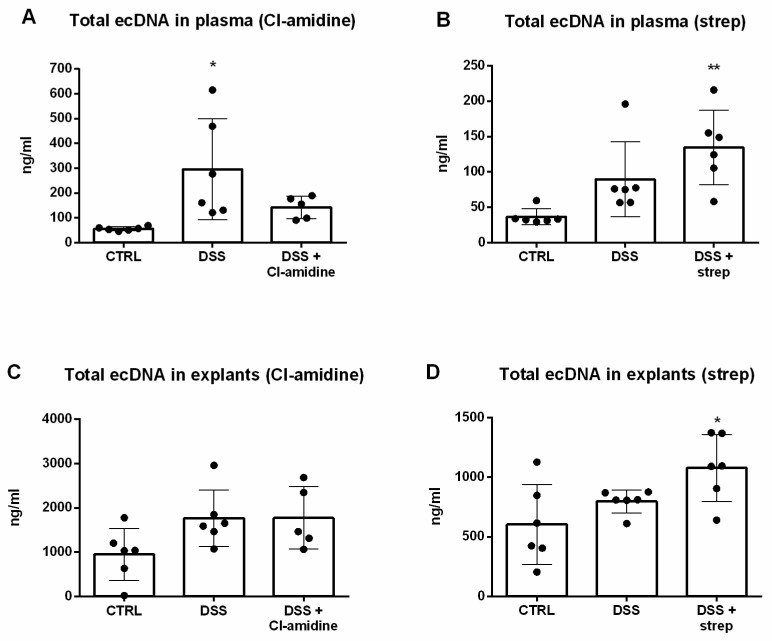
Cl-amidine treatment led to decrease of the concentration of total ecDNA in plasma (**A**), however this effect was not observed upon strep treatment (**B**). The concentration of total ecDNA in explants was not lower compared to positive control in either Cl-amidine (**C**) or strep (**D**) treatment. * = *p* < 0.05; ** = 0.05 > *p* > 0.01.

Next, in order to assess the effect of both inhibitors on the concentration of ecDNA, total ecDNA in plasma and explants was measured. While DSS group exhibited 5.3-fold higher total ecDNA in plasma concentration compared to CTRL, Cl-amidine managed to partially (2.5-fold higher compared to CTRL) decrease the concentration. The Cl-amidine-mediated lowering effect was pronounced enough to not reach statistical significance compared to CTRL group (*p* = 0.5, Figure 9A). Conversely, streptonigrin in DSS mice raised the concentration of total ecDNA in plasma 3.7-fold higher compared to CTRL (*p* = 0.004) and even 1.5-fold higher compared to DSS (*p* = 0.21, Figure 9B). Cl-amidine appeared to confer no effect to the concentration of total ecDNA in explants, since ecDNA concentration of the treated group was very similar to DSS group (*p* = 0.07, Figure 9C). In line with total ecDNA in plasma, streptonigrin further increased the concentration of total ecDNA in explants, reaching 1.8-fold increase compared to CTRL (*p* = 0.016) and even 1.35-fold increase compared to DSS (*p* = 0.18, Figure 9D).

## 4. Discussion

Our results have shown that the concentration of total ecDNA in plasma rises with increasing inflammation. This rise was accompanied by both increased endoscopic score and increased percentage of neutrophils undergoing NETosis. In addition, the rise in total ecDNA both in plasma and explants was found to correlate with the percentage of NETosis. These results prove the hypothesis that the concentration of total ecDNA increases in the murine model of chemically induced colitis. DNase activity in plasma remained relatively unchanged during the entire experiment, however in explants it seemed the activity was increasing with progression of inflammation. Despite this increasing trend, DNase activity appeared to be lower in the distal colon on day seven. Administration of PAD4 inhibitors, Cl-amidine and streptonigrin, did not lead to alleviation of intestinal inflammation according to relative weight and colon length, however endoscopic examination showed partial improvement.

Little is known about the dynamics of total ecDNA concentration either in plasma or colon during intestinal inflammation. Previously, our research group has shown that total ecDNA is higher on day seven of murine model of colitis [18]. To the best of our knowledge, this study is the first to demonstrate increasing concentration of total ecDNA in plasma at three timepoints. The concentration of total ecDNA appeared to be higher compared to CTRL group even in explants, although this difference did not reach statistical significance. Both ncDNA and mtDNA in plasma shared a similar trend of decreasing below the level of CTRL group until day five and rising slightly on day seven, however not significantly, probably due to high variability of data. This result was unexpected; while total ecDNA in plasma rises throughout colitis, absolute concentration of its subtypes decline. On the other hand, the relative abundance of ncDNA rises towards the more severe disease, as was shown on mapped reads analysis. This suggest that ncDNA is the subtype which possible mediates the downstream effects of ecDNA. Interestingly, the relative (but not absolute) dominance of this subtype is crucial.

As opposed to plasma, both ncDNA and mtDNA in explants unexpectedly reached the highest concentration on day three and decreased to the end of experiment. The rising concentration of total ecDNA in more severe colitis may be explained as damage to the intestinal epithelial barrier beginning on day three of DSS ingestion leading to higher intestinal permeability and more molecules to potentiate the inflammation (such as ecDNA) being able to cross the barrier. However, the tissue origin of the circulating ecDNA has not been analyzed and, thus, is not clear whether the damaged colonic tissue is the source of increased ecDNA concentrations. We speculate that initial phases of colitis lead to immediate damage of colonic cells and release of intracellular components in the environment, as was shown on increased ncDNA and mtDNA levels in colon explant medium. On the other hand, prolonged duration of pathological insult (ingestion of DSS) increases gut permeability leading to translocation of the released compounds into the circulation (instead of the environment).

In this regard, it needs to be noted, though, that DNase activity in explants showed a rising trend with the highest activity on day seven, it can be therefore assumed that this activity contributed at least in part to the observed phenomenon of decreasing ncDNA and mtDNA in explants on day seven. DNase activity is in agreement with the dynamics of total ecDNA concentration in plasma. Since throughout the experiment worsening of inflammation occurs, the concentration of total ecDNA in plasma may rise as a consequence of lower DNase activity, particularly on day seven. It appears that in CD patients, lower DNase activity, either as a cause or a result of inflammation, could promote and sustain inflammation [38,41].

The fragment analysis of total plasma ecDNA showed no pronounced difference among the groups, although an obvious trend was seen in PCA clustering from CTRL to DSS7 group. This indicates that fragmentation pattern of plasma ecDNA does not play a crucial role in the pathogenesis of DSS-induced colitis. Nevertheless, the proportion of mapped nuclear reads relative to total mapped reads increased with the severity of the disease in the expense of mitochondrial, bacterial and viral reads. Interestingly, the decrease in viral mapped reads is least pronounced compared with mitochondrial and bacterial, possibly due to the very low concentration of viruses in the circulation.

Even though there is evidence for both Cl-amidine and streptonigrin acting as inhibitors of NET formation in both murine models of colitis and human IBD patients [34,36,37,39], our results are not in agreement. This can be explained by the different dosing regimen used in our study, in which the inhibitors were only administered for five days of the seven-day long experiment. This way we aimed at determination of the effect of early intervention. Interestingly, despite both inhibitors did not seem to affect animal body weight or colon length, animals treated with either Cl-amidine or streptonigrin exhibited lower endoscopy score. Furthermore, although Cl-amidine treatment successfully lowered total ecDNA in plasma on day seven, this amelioration did not translate into alleviation of intestinal inflammation.

Colon mucosa immunostaining proved that PAD4 as a marker of NETs accumulates in the colonic mucosa in the second half of the experiment corresponding to more severe phases of colitis. Thus, it is clear that inhibition of this enzyme could possibly provide more therapeutic effect when performed in the latter stages of colitis corresponding to days four to seven of DSS consumption. This has not been shown herein, and thus represents one of the limitations of the study.

As mentioned, citrullination is important for NET formation [31]. Indeed, blockage of this post translational protein modification could provide an effective treatment and although the biological reason behind citrullination remains poorly characterized, it has been shown to be important for transcriptional regulation of gene expression [40,42,43,45]. Therefore, since long-term inhibition of citrullination on the level of the entire organism has not been studied, such prolonged therapy could cause other undesirable side effects. DNases offer a possible solution, although it is important to note that their activity and efficiency vary enormously in each laboratory animal, let alone human. This would advocate for a personalized medicine approach. Moreover, since the half-life of DNases in blood and colon tissue is also questionable and likely varies dramatically as well, additional studies need to be conducted in order to ascertain the possibility of such a venture. Systemic administration of DNase I was previously proved partially effective in ameliorating DSS colitis by decreasing inflammatory markers [44,47] and more recently by dissolving NETs [26]. In the view of our results, local intracolonic administration of DNase might represent an interesting way to digest ecDNA released from colonic cells into the cellular environment, which possibly reflects the pathophysiology of the initial phases of the disease.

The role of circulating ecDNA in the pathogenesis of inflammatory bowel disease was recently summarized in a comprehensive review published by our group [46,48]. However, there is still a number of molecular phenomena that need to be uncovered to fully ascertain the mechanisms by which ecDNA and DNases modulate the disease activity. The current study provided a deeper insight on the dynamics of ecDNA concentration, fragmentation and origin throughout the course of the disease and opened new avenues for future research. These include determination of the stimuli which lead to formation of NETs and exploration of the effect of free circulating ecDNA on pathways of innate immunity. Last, but not least, the therapeutic potential of modulating the ecDNA pathways, including the use of DNases, needs to be rigorously addressed in future studies.

## Figures and Tables

**Table 1:** Endoscopy scoring system. The table describes score values of each examined category.

Category	Score	Description
Translucency	0	Vascularization fully visible
	1	Vascularization partially visible
	2	Vascularization not visible
Fibrin	0	No fibrin is present in the mucosa
	1	Small fibrin deposits in the mucosa
	2	Large fibrin deposits in the mucosa
Bleeding	0	No bleeding
	1	Several sites of mucosal bleeding
	2	Many sites of mucosal bleeding, may obstruct camera of the endoscope, bleeding may start spontaneously or as a reaction to contact with endoscope, blood may directly flow out of the anus
	3	Profound mucosal bleeding, usually obstructs camera of the endoscope, bleeding often starts spontaneously and blood usually flows out of the anus
Reddening	0	No reddening visible
	1	Several sites of mucosal reddening
	2	Many sites of mucosal reddening

## Data Availability

Data available on request due to restrictions.

## Supplementary Materials

The following are available online at https://www.mdpi.com/2073-4409/10/1/81/s1, Figure S1: Representative plots of cell sorting, Figure S2: Markers of disease activity, Figure S3: Representative images of endoscopic examination.
