# Supplementary material for: Extracellular DNA Correlates with Intestinal Inflammation in Chemically Induced Colitis in Mice
Source: Cells. 2021 Jan 6;10(1):81. doi: 10.3390/cells10010081 (PMC7825321; doi:10.3390/cells10010081)
Supplement: Supplementary file 1 [file cells-10-00081-s001.pdf]

Supplementary Material

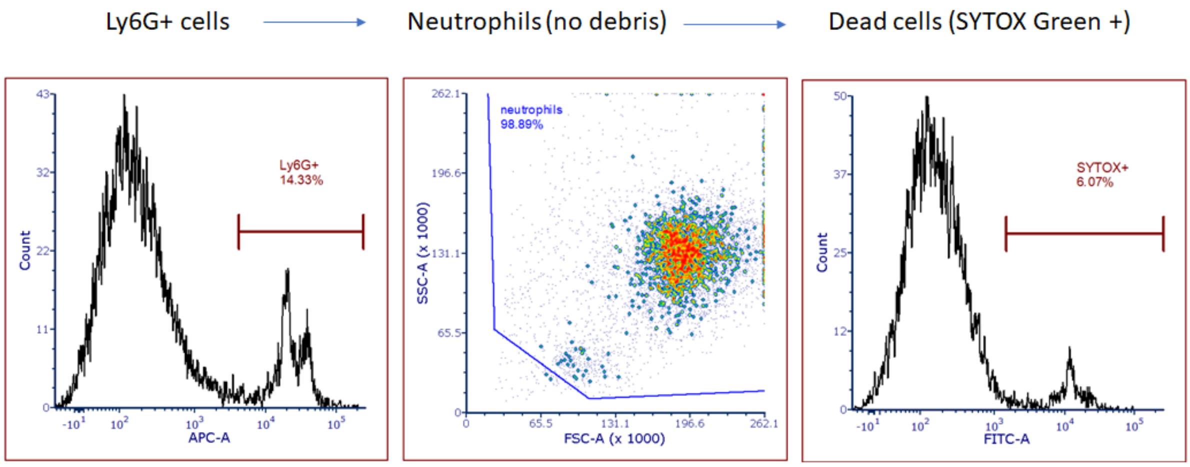

Figure S1. Representative plots of cell sorting.

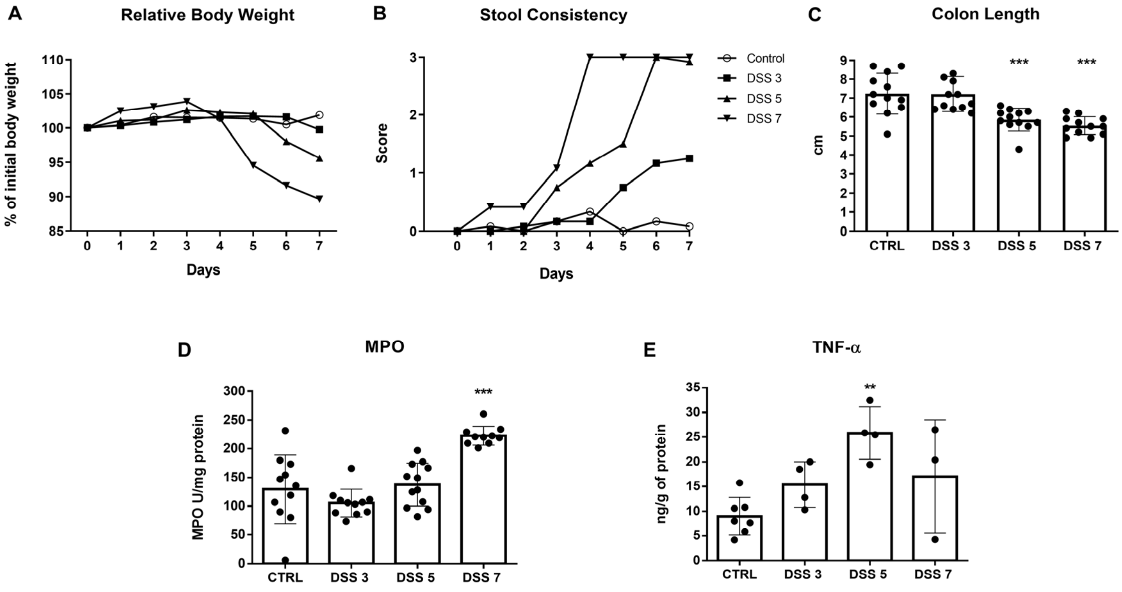

Figure S2. Markers of disease activity (**A**) ingestion of 3% dextran sulfate sodium (DSS) decreased relative body weight of all experimental groups from day 3. (**B**) higher stool consistency score of all experimental groups confirmed extensive intestinal inflammation compared to control group. (**C**) colon length of DSS 5 and DSS 7 groups was significantly shorter compared to control group. (**D**) MPO activity was significantly higher in DSS 7 group compared to control group. (**E**) TNF-α showed a rising trend up to day 5 reaching statistical significance and decreased on day 7. ** = $0.05 > p > 0.01$; *** = $p < 0.001$.

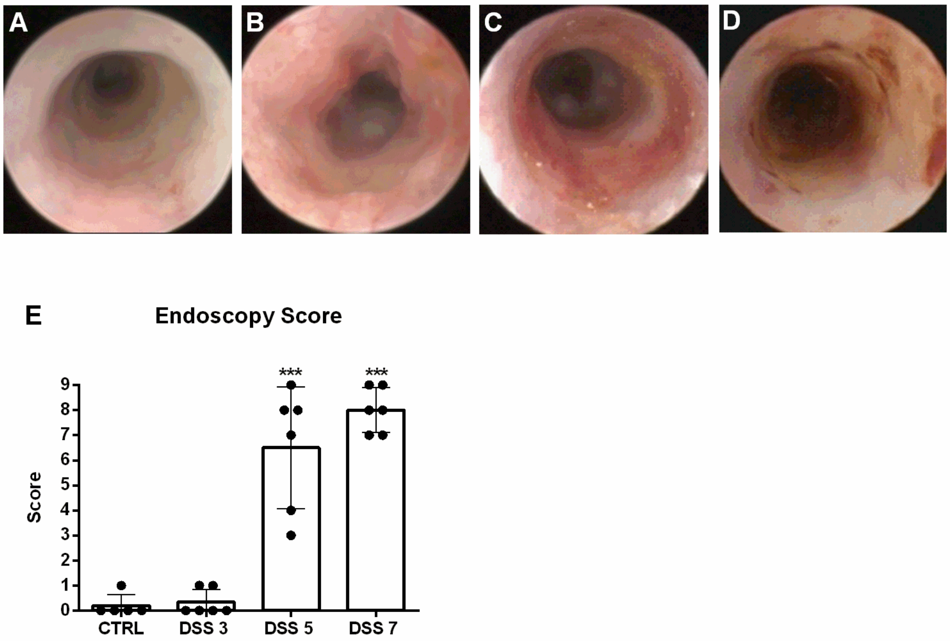

Figure S3. Representative images of endoscopic examination of (**A**) control group, (**B**) DSS 3, (**C**) DSS 5 and (**D**) DSS 7. (**E**) endoscopy score confirmed significant intestinal inflammation in DSS 5 and DSS 7 groups. [M1] *** = $p < 0.001$.
